# Factors Associated with Extraction following Root Canal Filling in Adults

**DOI:** 10.1177/0022034520982962

**Published:** 2021-01-05

**Authors:** H. Fransson, L. Bjørndal, F. Frisk, V.S. Dawson, K. Landt, P.-E. Isberg, EndoReCo, T. Kvist

**Affiliations:** 1 Department of Endodontics, Malmö University, Malmö, Sweden; 2 Department of Endodontology, University of Gothenburg, Gothenburg, Sweden; 3 Section of Clinical Oral Microbiology, Cariology and Endodontics, University of Copenhagen, Copenhagen, Denmark; 4 Institute for Postgraduate Dental Education, Jönköping, Sweden; 5 Department of Statistics, Lund University School of Economics and Management, Lund University, Lund, Sweden; 6 Endodontic Research Collaboration in Scandinavia

**Keywords:** demography, endodontics, permanent dental restoration, root canal therapy, tooth extraction

## Abstract

Essentially, root fillings are performed to preserve natural teeth. Over time, however, some root-filled teeth will inevitably be extracted. The aim of this historical prospective cohort study in the adult Swedish population was to identify factors associated with extractions within 5 y of registration of a root filling. The cohort consisted of all those whose root fillings had been reported to the tax-funded Swedish Social Insurance Agency (SSIA) in 2009. Demographic data on the individuals registered with a root filling (sex, age, country of birth, disposable income, educational level, and marital status) were received from Statistics Sweden or the SSIA. Dental care setting, tooth type, and any registration of subsequent restorations within 6 mo were received from the SSIA. Multivariable regression analysis was used, and *P* < 0.05 was considered statistically significant. In total, 216,764 individuals had been registered with at least 1 root filling. Individuals (*n* = 824) without complete data were excluded from the analyses. After 5 y, 9.3% of the root-filled teeth had been registered as extracted. Logistic regression analysis found significant associations for all variables except country of birth, disposable income, and educational level. The highest odds ratios for extractions were associated with the type of restoration: teeth with no registration of any restoration and teeth with a direct restoration combined with a post were 3 times more likely to undergo extraction than teeth restored with an indirect restoration combined with a post and core. Overall, high odds ratios for extractions were associated with any type of composite restoration, including composite fillings and crowns combined with or without any post. In summary, after root filling in the Swedish adult population, several individual- and tooth-specific variables were associated with extraction. The reasons for the extractions remain to be studied further.

## Introduction

Due to improved oral health in Western countries (Norderyd et al. 2015; Stock et al. 2016), teeth are now more likely to be retained throughout a person’s life. This may be due to a lower burden of oral disease but also to tooth-preserving treatments such as root canal treatment (RCT; Bjørndal et al. 2006). By preventing or eliminating an infection in the root canal, an RCT is intended to avoid extraction and maintain the standing dentition. Generally, considerable satisfaction is reported by individuals who recently had an RCT (Wigsten et al. 2020). The proportion of root canal–treated teeth surviving >2 to 10 y ranges from 74% to 94% (Lumley et al. 2008; Ng et al. 2010; Raedel et al. 2015). It has been proposed that tooth survival after RCT depends both on the type of restoration that is placed after the treatment and on the type of teeth (Ng et al. 2010; Landys Borén et al. 2015). A focus on demographic factors such as sex, age, disposable income, and educational level (Lumley et al. 2008; Ng et al. 2011; Landt et al. 2018) also highlights the influence of socioeconomic factors on treatment outcome (Collares et al. 2018).

Traditionally, clinical research on the outcome of RCT procedures has focused mainly on the ability of RCT to preserve or reestablish healthy periapical tissues. In the last 20 y, however, increasing attention has been paid to tooth survival as a central outcome measure. This shift in focus is likely to have been driven partly by the successful establishment of single implants (Albrektsson and Wennerberg 2005) as a feasible alternative to RCT. As there is often a choice of treatment—either keeping the natural tooth through an RCT or undergoing an extraction and replacement—patients, dentists, and third-party stakeholders would all benefit from better evidence on the risk factors for loss of root-filled teeth (Iqbal and Kim 2008; Setzer and Kim 2014). A comprehensive systematic review concluded that the evidence on the effect of prognostic factors on tooth survival was weak, often because of small sample sizes (Ng et al. 2010). To make it possible to predict which teeth had a high risk for extraction, subsequent studies (Ng et al. 2011; Landys Borén et al. 2015; Raedel et al. 2015) indicated a need for studies on prognostic factors. For this reason, we aimed to identify factors in the adult Swedish population that were associated with the extraction of teeth within 5 y of a registered root filling.

## Materials and Methods

We followed the STROBE guidelines (Strengthening the Reporting of Observational Studies in Epidemiology; von Elm et al. 2014).

### Data Collection

Data collection for this study has been described previously (Fransson et al. 2016; Landt et al. 2018). From the Swedish Social Insurance Agency (SSIA), we received data on root fillings that had been registered, and thus completed, between January 1 and December 31, 2009, by searching for registration codes for root filling (501 to 504). Because the codes are registered for a specific tooth in a specific individual, longitudinal studies are possible, and because all adult citizens are insured by the SSIA, data are available for the whole population from the age of 20 y. As dentists acquire reimbursements directly by registering a code used in conjunction with a treatment (e.g., root filling), practically all dentists in Sweden are associated with the SSIA. The insurance is based on a high-cost scheme that, irrespective of an individual’s finances, uses a stepwise scale to subsidize higher proportions of costlier treatments (Dental and Pharmaceutical Benefits Agency 2017).

We selected the outcome measure “tooth extraction” for teeth registered as root filled in 2009 by searching the codes for extraction (401 to 404) in the subsequent 5 y. Information on different types of restorations was collected by searching the SSIA register for the codes used for direct coronal restoration (701 to 707) and indirect coronal restoration, indirect or direct post, and core (801 to 807). Data on demographic variables representing different aspects of an individual’s socioeconomic status (disposable income, educational level, marital status, and country of birth) had been collected from Statistics Sweden, another government agency that issues official statistics.

### Independent Variables

Data were collected on each individual’s sex (male, female); age, divided into 5 categories (20 to 29, 30 to 49, 50 to 64, 65 to 74, ≥75 y); country of birth (Sweden or elsewhere); disposable income (10,000 SEK per year [Swedish crowns], equivalent to US $1,020 based on the exchange rate on October 17, 2019 [1 USD = 9.80 SEK]); and highest attained educational level, divided into 6 categories (primary school <9 y, primary school >9 y, upper secondary <2 y, upper secondary school >2 y, postgraduate studies, unknown). Marital status was also recorded and divided into 4 categories (single, married or registered partner, divorced or divorced partner, widow or widower).

The dentists who registered root fillings were divided into type of dental practice setting (private, public, or dental school). Tooth type was divided into 6 categories (maxillary molar, premolar, or canine/incisor and mandibular canine/incisor, premolar, or molar).

In different combinations, we analyzed the following types of coronal restoration that had been registered within 6 mo of registration of root filling: indirect coronal restorations and direct coronal restorations, divided into composite filling and composite crown, indirect post and core, and direct post and core (Table 1). If a tooth was registered with several coronal restorations during this 6-mo period, any registration of an indirect restoration superseded the previous direct restoration.

**Table 1.** Characteristics of the Cohort: Total Root-Filled Teeth Registered and Extracted.

Variable	Total	Extracted
Sex		
Male	108,902 (50.2)	9,978 (9.2)
Female	107,862 (49.8)	10,277 (9.5)
Age group, y		
20 to 29	13,337 (6.1)	802 (6.0)
30 to 49	63,241 (29.2)	5,499 (8.7)
50 to 64	77,735 (35.9)	7,603 (9.8)
65 to 74	40,141 (18.5)	4,151 (10.3)
≥75	22,310 (10.3)	2,200 (9.9)
Country of birth^a^		
Born in Sweden	164,063 (83.5)	17,091 (9.4)
Born abroad	32,426 (16.5)	3,164 (8.9)
Educational level^b^		
Primary school <9 y	50,212 (23.3)	4,685 (9.3)
Primary school >9 y/upper secondary	99,356 (46.0)	9,344 (9.4)
Upper secondary school <2 y	17,492 (8.1)	1,546 (8.8)
Upper secondary school >2 y	44,852 (20.8)	4,272 (9.5)
Postgraduate studies	2,270 (1.0)	244 (10.7)
Unknown	1,773 (0.8)	150 (8.5)
Marital status^b^		
Single	55,398 (25.6)	4,827 (8.7)
Married or registered partner	114,356 (52.0)	10,549 (9.2)
Divorced or divorced partner	32,824 (15.2)	3,499 (10.7)
Widow/widower	13,377 (6.2)	1,366 (10.2)
Dental care setting		
Public	72,881 (33.6)	6,179 (8.5)
Private	143,368 (66.1)	14,033 (9.8)
School	515 (0.3)	43 (8.3)
Tooth group		
Maxillary molar	45,692 (21.1)	5,166 (11.3)
Maxillary premolar	39,977 (18.4)	3,128 (7.8)
Maxillary canine or incisor	34,065 (15.7)	2,731 (8.0)
Mandibular molar	55,173 (25.4)	6,237 (11.3)
Mandibular premolar	27,871 (12.9)	1,772 (6.4)
Mandibular canine or incisor	13,986 (6.6)	1,221 (8.7)
Restoration within 6 mo		
No restoration	42,275 (19.5)	5,633 (13.3)
Composite filling	109,840 (50.7)	10,425 (9.5)
Composite filling and direct post and core	562 (0.2)	81 (14.4)
Composite crown	19,207 (8.9)	1,903 (9.9)
Composite crown and in direct post and core	278 (0.1)	31 (11.1)
Indirect coronal restoration	11,147 (5.1)	569 (5.1)
Indirect coronal restoration and direct post and core	13,147 (6.1)	699 (5.3)
Indirect coronal restoration and indirect post and core	20,308 (9.4)	914 (4.5)

Consisting of all teeth registered as root filled over 1 y in Sweden and the frequency of root-filled teeth registered as extracted during the following 5 y. Values are presented as *n* (%).

a Missing data for 20 individuals.

b Missing data for 809 individuals.

### Statistical Analysis

SPSS 22 (IBM) was used to perform statistical analyses. The distribution of tooth-specific and demographic factors is described with descriptive data and frequencies. Multivariate logistic regression analysis was used to analyze associations between tooth-specific and demographic variables, with the dependent variable being extraction of the root-filled tooth. Odds ratio (OR) estimates were presented with a 95% CI. The level of significance was set at 0.05.

### Ethical Issues

The study was approved by the Regional Ethical Board at Lund University, Sweden (Dnr 211/800).

## Results

The SSIA database for 2009 contained registrations for 248,299 root fillings in 217,047 individuals. Per individual, we included only the first root-filled tooth in our analyses. As some individuals had missing data and were excluded, the data were based on 216,764 individuals and the logistic regression on 215,940 individuals. A total of 20,255 (9.3%) teeth that had been root filled in 2009 were subsequently registered as having been extracted during the subsequent 5-y period.

The distribution of the variables analyzed in the cohort of root-filled individuals showed the following: there was an equal frequency of men and women; the 50- to 64-y age group was the most frequent; having been born in Sweden was more frequent than having been born abroad; being married or in a registered partnership was the most frequent marital status; private practice was the most frequent dental care setting; mandibular molars were the most frequent tooth group; and composite filling was the most frequent type of restoration (Table 1). Although the extraction frequencies for most variables differed by only a few percent, the frequency of extractions per type of restoration ranged from 4.5% (indirect coronal restoration in combination with an indirect post and core) to 14.4% (composite filling and direct post and core). Molars accounted for 56.3% of extracted teeth and 45.5% of nonextracted teeth. Similarly, within 6 mo of completion of root filling, no restorations were registered for 27.8% of extracted teeth versus 18.6% for nonextracted teeth.

The multivariate regression analysis found significant associations between sex, age group, marital status, caregiver, tooth group, and type of restoration and extraction of the root-filled teeth (all *P <* 0.001, except for sex, where *P* = 0.002 (Table 2). The highest OR for extractions applied to 3 variables: age group, tooth group, and type of restorations. The odds (95% CI) for extraction in the 65- to 74-y age group (2.186; 2.007 to 2.446) and for individuals ≥75 y (2.227; 2.027 to 2.446) were more than double those for individuals in the 20- to 29-y age group. Similarly, the odds for extraction of root-filled maxillary molars (1.948; 1.839 to 2.063) and mandibular molars (1.971; 1.863 to 2.084) were almost double those for root-filled mandibular premolars.

**Table 2.** Logistic Regression Analysis of Factors Associated with the Root-Filled Teeth Registered as Extracted during the 5-y Study Period.

Variable	Odds Ratio	95% CI	*P* Value
Sex			0.002^a^
Male	Ref		
Female	1.049	1.018 to 1.081	
Age group, y			<0.001^a^
20 to 29	Ref		
30 to 49	1.511	1.397 to 1.634	<0.001
50 to 64	1.910	1.763 to 2.079	<0.001
65 to 74	2.186	2.007 to 2.446	<0.001
≥75	2.227	2.027 to 2.446	<0.001
Country of birth			0.123^a^
Born abroad	Ref		
Born in Sweden	0.967	0.928 to 1.009	
Disposable income^b^	1.00	0.999 to 1.000	0.720^a^
Educational level			0.154^a^
Primary school <9 y	Ref		
Primary school >9 y/upper secondary	1.011	0.973 to 1.050	0.587
Upper secondary school <2 y	0.952	0.894 to 1.012	0.116
Upper secondary school >2 y	0.977	0.933 to 1.022	0.307
Postgraduate studies	1.087	0.947 to 1.248	0.237
Unknown	0.950	0.799 to 1.130	0.562
Marital status			<0.001^a^
Married or registered partner	Ref		
Single	1.056	1.016 to 1.098	0.006
Divorced or divorced partner	1.182	1.134 to 1.231	<0.001
Widow/widower	1.074	1.006 to 1.147	0.031
Dental care setting			<0.001^a^
School	Ref		
Private	1.469	1.072 to 2.013	0.014
Public	1.257	0.916 to 1.724	0.138
Tooth group			<0.001^a^
Mandibular premolar	Ref		
Maxillary molar	1.948	1.839 to 2.063	<0.001
Maxillary premolar	1.359	1.278 to 1.444	<0.001
Maxillary canine or incisor	1.359	1.276 to 1.447	<0.001
Mandibular molar	1.971	1.863 to 2.084	<0.001
Mandibular canine or incisor	1.274	1.179 to 1.376	<0.001
Restoration within 6 mo			<0.001^a^
Indirect coronal restoration and indirect post and core	Ref		
No restoration	3.268	3.038 to 3.515	<0.001
Composite filling	2.246	2.092 to 2.411	<0.001
Composite filling and direct post and core	3.209	2.509 to 4.102	<0.001
Composite crown	2.201	2.026 to 2.391	<0.001
Composite crown and direct post and core	2.595	1.773 to 3.798	<0.001
Indirect coronal restoration	1.085	0.974 to 1.209	0.139
Indirect coronal restoration and direct post and core	1.155	1.044 to 1.278	0.005

Logistic regression analysis was used to analyze associations between tooth/individual variables and the extraction of the root-filled tooth. Nagelkerke *r*^2^ = 0.036, McFadden *r*^2^ = 0.027.

Ref, reference.

a Likelihood ratio test for the total variable.

b Odds ratio per 10,000 SEK–increment in income (Swedish crowns).

Teeth restored (within 6 mo) with indirect coronal restoration had the lowest risk for extraction regardless of whether the indirect coronal restoration was combined with an indirect post or not (*P* = 0.139). However, if the indirect coronal restoration was combined with a direct post, the OR (95% CI) for extraction was higher (1.155; 1.044 to 1.278). For teeth restored (within 6 mo) with any type of composite restoration, regardless of whether it was combined with a post or not, the OR for extraction was higher, varying from 2.2 to 3.2. The highest risk for extraction was observed for teeth without a registered permanent restoration (3.26; 3.038 to 3.515). For teeth with no registration of any restoration, the OR for extraction was 3.268 (3.038 to 3.515).

## Discussion

The main finding of our study of factors associated with extraction following root canal filling in the adult Swedish population is that while there were associations for sex, age group, marital status, dental care setting, tooth group, and restoration types, there were no associations for country of birth, disposable income, or educational level and the registration of extraction. The highest risk for extraction was found for older age groups, molars, and teeth with no registration of a coronal restoration or teeth restored with composite.

A considerable majority of the root fillings and extractions in this study were performed by general dentists in private practice or in the public dental health service. As dental care in Sweden is totally tax funded for all children and adolescents up to the age of 20 y, this age group is not reported to the SSIA and was not therefore included in our study. Otherwise, the data that we present represent nearly all root fillings completed in Sweden during 2009 and any subsequent restorative procedures and extractions of the same teeth for a postoperative period of 5 to 6 y (Fransson et al. 2016). External validity can be assumed to other countries in which general dentists perform the major proportion of endodontic treatments. With regard to external validity, it should also be remarked that the oral health care provided in a given country will be influenced not only by factors such as the prevailing system for reimbursing dental care but also by the population’s burden of disease. Overall, the oral health of the Swedish population is regarded as good: the number of decayed, missing, and filled teeth in 12-y-olds is very low (0.0 to 1.1; FDI 2015), and cohorts of 60- and 70-y-olds in a cross-sectional study were also shown to have almost complete dentition, in which a mean 6.7 teeth were root filled (Norderyd et al. 2015).

Overall, 9.3% of the root-filled teeth were extracted during the subsequent 5-y period, and we previously reported that the extractions cumulate in an almost linear fashion (Fransson et al. 2016). The proportion of extractions of root-filled teeth is in concordance to what has been documented from other countries (Lumley et al. 2008; Ng et al. 2010; Raedel et al. 2015).

A study conducted in England and Wales demonstrated lower survival of root fillings in people who were entitled to additional financial support for their dental care (Lumley et al. 2008). However, our results regarding disposable income or country of birth and educational level were not associated with extraction. As we cannot identify patients in our database who have received extra financial support in addition to their general dental insurance, we cannot evaluate whether the survival of root fillings that were part financed by social authorities or charity is better or worse. Although it is conceivable that people with a lower socioeconomic status who need endodontic treatment will chose extraction over RCT, it was not possible to study this in the available registry. However, those in our study who were registered as single, divorced, or widow/widower were more likely to have the root-filled tooth extracted. For this reason, appropriate methods should be used to further investigate the potential influence of socioeconomic variables on treatment decisions involving endodontically involved teeth and the outcome of RCT.

A recent systematic review evaluating the influence of increased patient age on longitudinal outcomes assessed on radiographs (i.e., the presence of apical periodontitis) concluded that increased patient age did not reduce the success rate of nonsurgical RCT (Shakiba et al. 2017). Age is nonetheless known to influence the risk for extraction of root-filled teeth (Lumley et al. 2008; Landys Borén et al. 2015). Intuitively, associations between age and the extraction of a root-filled tooth are natural consequences of aging, as such a tooth has been functioning for a longer period and is likely to have less remaining tooth structure, thereby affecting its restorability (Afrashtehfar et al. 2017). Older individuals are also more likely to have other conditions, such as marginal periodontitis, that might be a reason for extraction (Petersson et al. 2016). Extractions may also be due to wider treatment needs; it has even been speculated that the Swedish insurance system drives extractions and replacement with fixed bridges or implants (Dental and Pharmaceutical Benefits Agency 2017).

Most root fillings were performed in the private sector or in the public dental health sector. Teeth root filled in the private sector were more frequently extracted than in the public dental health sector. However, we have been unable to identify any studies on the influence of the type of dental care setting and tooth extraction.

Our finding that tooth type was associated with extractions is consistent with earlier reports that root-filled molars are at a higher risk for extractions (Ng et al. 2010; Landys Borén et al. 2015; Petersson et al. 2016). However, it conflicts with a German study based on insurance company data on half a million teeth, which showed very little difference in survival between multirooted and single-rooted teeth. This may be attributable to a selection process where only molar teeth judged to have a good prognosis were root filled, as the German insurance system has strict criteria for reimbursing endodontic treatment of multirooted teeth (Raedel et al. 2015).

The data that we present in this study are derived from registries that contain no information on pulp or periapical diagnosis, periodontal status, or any existing restorations. However, another recent study on public dental health care in Sweden showed that the majority of teeth in which RCT was initiated had previously been restored and had undergone considerable loss of tooth substance. The same study showed that the most commonly registered indication was pulpal necrosis with apical periodontitis, followed by pulpitis. Retreatment was seldom performed (Wigsten et al. 2019). These findings were similar to those of a Danish questionnaire survey of general dental practitioners (Bjørndal et al. 2006).

Considerable attention has been focused on the impact of the type of restoration that is placed after root filling (Ng et al. 2010). The importance of the coronal restoration to establishing periapical health is probably due to its ability to act as a barrier against coronal leakage (Dawson et al. 2016). Evaluation of tooth survival suggests that the ability of the coronal restoration to enhance the tooth’s ability to withstand masticatory forces is of primary importance. Although we were unable in our study to differentiate among full crown, onlay, and inlay within the group of indirect coronal restorations, a large proportion were probably full crowns, as these were the indirect coronal restorations found most in the most recent Swedish epidemiologic study (Dawson et al. 2016). Another issue is that even though a root-filled tooth has been registered with a direct restoration after completion of the root filling, the root might have been filled through an existing full crown. It is quite remarkable that at 6 mo after root filling, almost one-fifth of the teeth (19.5%) had not yet been registered with any restoration. We can only speculate about the reasons. Several dentists may apply a precautionary principle—that is, to provide a restoration when symptoms subsided completely or even when radiographic signs of healing appear. Consequently, many teeth may have received a permanent restoration after 6 mo. However, to study the influence of placement of a permanent restoration, a cutoff time point was needed.

The high OR that we found for extractions without registration of an indirect coronal restoration does not allow us to make immediate recommendations on the choice of restorations. Since we are studying a historical cohort, no randomization has been performed, and the dentist may have chosen to place direct restorations on teeth judged to have an unfavorable prognosis based on factors unavailable to us. Account should also be taken of the issue involving the costs associated with different restorations (Wigsten et al. 2018). Most teeth in this study were not restored with an indirect restoration. Instead, on the basis of their clinical experience with factors such as periodontal status and remaining tooth substance, the treating dentists decided which teeth should be considered for an indirect restoration.

No registration of any restoration within 6 mo was associated with a higher risk for extraction (OR, 3.268; 95% CI, 3.038 to 3.515; *P* < 0.001), a finding that was almost equal to teeth restored with a composite filling in combination with a post and core (3.209; 2.509 to 4.102; *P* < 0.001). As few teeth were registered with a composite filling in combination with a post and core, we speculate that the combination was used for teeth judged to have dubious prognosis. A composite filling alone was associated with a risk of extraction (2.246; 2.092 to 2.411; *P <* 0.001) that was as high as that for a composite crown (2.201; 2.026 to 2.391; *P* < 0.001). Overall, the OR for extraction was higher for a direct post and core than for an indirect post and core. A 20-y follow-up study of root-filled teeth found that prefabricated posts other than screw posts were one of several risk factors associated with a low OR of tooth survival (Petersson et al. 2016). Randomized controlled studies should now build on this by establishing which type or types of restoration provide the best survival of root-filled teeth in different clinical situations (Ferrari et al. 2012).

In conclusion, despite the limitations related to this registry study where the actual reasons for extractions cannot be ascertained, the findings implicate that dentists planning for RCT should consider age, tooth group, and the type of restoration. These variables had the strongest associations for extraction 5 y after root filling, whereas socioeconomic indicators were not associated with extraction. In particular, when root fillings are performed in molar teeth in older individuals, clinicians should pay attention to their choice of restoration to promote tooth retention over time.

## Author Contributions

H. Fransson, contributed to conception, design, data acquisition, analysis, and interpretation, drafted and critically revised the manuscript; L. Bjørndal, F. Frisk, V.S. Dawson, T. Kvist, contributed to conception, design, data acquisition, and interpretation, critically revised the manuscript; K. Landt, contributed to data acquisition and interpretation, drafted and critically revised the manuscript; P.-E. Isberg, contributed to data acquisition and analysis, critically revised the manuscript. All authors gave final approval and agree to be accountable for all aspects of the work.

## References

[bibr1-0022034520982962] Afrashtehfar KI Ahmadi M Emami E Abi-Nader S Tamimi F. 2017. Failure of single-unit restorations on root filled posterior teeth: a systematic review. Int Endod J. 50(10):951–966. 27870102 10.1111/iej.12723

[bibr2-0022034520982962] Albrektsson T Wennerberg A. 2005. The impact of oral implants—past and future, 1966–2042. J Can Dent Assoc. 71(5):327. 15949251

[bibr3-0022034520982962] Bjørndal L Laustsen MH Reit C. 2006. Root canal treatment in Denmark is most often carried out in carious vital molar teeth and retreatments are rare. Int Endod J. 39(10):785–790. 16948664 10.1111/j.1365-2591.2006.01149.x

[bibr4-0022034520982962] Collares K Opdam NJ Peres KG Peres MA Horta BL Demarco FF Correa MB. 2018. Higher experience of caries and lower income trajectory influence the quality of restorations: a multilevel analysis in a birth cohort. J Dent. 68:79–84. 29169969 10.1016/j.jdent.2017.11.009

[bibr5-0022034520982962] Dawson VS Petersson K Wolf E Åkerman S. 2016. Periapical status of root-filled teeth restored with composite, amalgam, or full crown restorations: a cross-sectional study of a Swedish adult population. J Endod. 42(9):1326–1333. 27452294 10.1016/j.joen.2016.06.008

[bibr6-0022034520982962] Dental and Pharmaceutical Benefits Agency. 2017. Dental care benefits scheme 2017. Stockholm (Sweden): Dental and Pharmaceutical Benefits Agency.

[bibr7-0022034520982962] Ferrari M Vichi A Fadda GM Cagidiaco MC Tay FR Breschi L Polimeni A Goracci C. 2012. A randomized controlled trial of endodontically treated and restored premolars. J Dent Res. 91(7):72S–78S. 22699672 10.1177/0022034512447949

[bibr8-0022034520982962] Fransson H Dawson VS Frisk F Bjørndal L EndoReCo, Kvist T. 2016. Survival of root-filled teeth in the Swedish adult population. J Endod. 42(2):216–220. 26813417 10.1016/j.joen.2015.11.008

[bibr9-0022034520982962] Iqbal M Kim S. 2008. A review of factors influencing treatment planning decisions of single-tooth implants versus preserving natural teeth with nonsurgical endodontic therapy. J Endod. 34(5):519–529. 18436028 10.1016/j.joen.2008.01.002

[bibr10-0022034520982962] Landt K Hagstam-Harrison L Kvist T Frisk F Dawson VS Bjørndal L EndoReCo, Fransson H. 2018. Demographic factors in Swedish adults undergoing root filling and subsequent extraction of a maxillary first molar: a comparative study. Int Endod J. 51(9):975–980. 29424077 10.1111/iej.12907

[bibr11-0022034520982962] Landys Borén D Jonasson P Kvist T. 2015. Long-term survival of endodontically treated teeth at a public dental specialist clinic. J Endod. 41(2):176–181. 25453569 10.1016/j.joen.2014.10.002

[bibr12-0022034520982962] Lumley PJ Lucarotti PS Burke FJ. 2008. Ten-year outcome of root fillings in the General Dental Services in England and Wales. Int Endod J. 41(7):577–585. 18479376 10.1111/j.1365-2591.2008.01402.x

[bibr13-0022034520982962] Ng YL Mann V Gulabivala K. 2010. Tooth survival following non-surgical root canal treatment: a systematic review of the literature. Int Endod J. 43(3):171–189. 20158529 10.1111/j.1365-2591.2009.01671.x

[bibr14-0022034520982962] Ng YL Mann V Gulabivala K. 2011. A prospective study of the factors affecting outcomes of non-surgical root canal treatment: part 2. Tooth survival. Int Endod J. 44(7):610–625. 21366627 10.1111/j.1365-2591.2011.01873.x

[bibr15-0022034520982962] Norderyd O Koch G Papias A Köhler AA Helkimo AN Brahm CO Lindmark U Lindfors N Mattsson A Rolander B, et al. 2015. Oral health of individuals aged 3-80 years in Jönköping, Sweden during 40 years (1973-2013): II. Review of clinical and radiographic findings. Swed Dent J. 39(2):69–86. 26529833

[bibr16-0022034520982962] Petersson K Fransson H Wolf E Håkansson J. 2016. Twenty-year follow-up of root filled teeth in a Swedish population receiving high-cost dental care. Int Endod J. 49(7):636–645. 26139565 10.1111/iej.12495

[bibr17-0022034520982962] Raedel M Hartmann A Bohm S Walter MH. 2015. Three-year outcomes of root canal treatment: mining an insurance database. J Dent. 43(4):412–417. 25676179 10.1016/j.jdent.2015.01.013

[bibr18-0022034520982962] Setzer FC Kim S. 2014. Comparison of long-term survival of implants and root canal treated teeth. J Dent Res. 93(1):19–26. 24065635 10.1177/0022034513504782 PMC3872851

[bibr19-0022034520982962] Shakiba B Hamedy R Pak JG Barbizam JV Ogawa R White SN. 2017. Influence of increased patient age on longitudinal outcomes of root canal treatment: a systematic review. Gerodontology. 34(1):101–109. 27198169 10.1111/ger.12231

[bibr20-0022034520982962] Stock C Jürges H Shen J Bozorgmehr K Listl S. 2016. A comparison of tooth retention and replacement across 15 countries in the over-50s. Community Dent Oral Epidemiol. 44(3):223–231. 26706945 10.1111/cdoe.12209

[bibr21-0022034520982962] von Elm E Altman DG Egger M Pocock SJ Gøtzsche PC Vandenbroucke JP; STROBE Initiative. 2014. The Strengthening the Reporting of Observational Studies in Epidemiology (STROBE) statement: guidelines for reporting observational studies. Int J Surg. 12(12):1495–1499.25046131

[bibr22-0022034520982962] Wigsten E Jonasson P EndoReCo, Kvist T. 2019. Indications for root canal treatment in a Swedish county dental service: patient- and tooth-specific characteristics. Int Endod J. 52(2):158–168. 30107035 10.1111/iej.12998

[bibr23-0022034520982962] Wigsten E Kvist T Dawson VS Isberg PE EndoReCo, Fransson H. 2018. Comparative analysis of general dental practitioners’ fees and scheduled fees for root canal treatment and coronal restorations in the adult population of Sweden: a 5-year follow-up of data from the Swedish Dental Register. Int Endod J. 51(2):141–147. 28708240 10.1111/iej.12817

[bibr24-0022034520982962] Wigsten E Kvist T Jonasson P EndoReCo, Davidson T. 2020. Comparing quality of life of patients undergoing root canal treatment or tooth extraction. J Endod. 46(1):19–28. 31843125 10.1016/j.joen.2019.10.012

[bibr25-0022034520982962] World Dental Federation. 2015. The challenge of oral disease—a call for global action by FDI World Dental Federation. Maps and graphics. Myriad Editions [accessed 2020 Aug 20]. https://www.fdiworlddental.org/resources/publications/oral-health-atlas/oral-health-atlas-2015

